# Aggravating Impact of Nanoparticles on Immune-Mediated Pulmonary Inflammation

**DOI:** 10.1100/tsw.2011.44

**Published:** 2011-02-14

**Authors:** Ken-Ichiro Inoue, Hirohisa Takano

**Affiliations:** ^1^ Department of Public Health and Molecular Toxicology, School of Pharmacy, Kitasato University, Tokyo, Japan; ^2^ Environmental Health Sciences Division, National Institute for Environmental Studies, Tsukuba, Japan

**Keywords:** nanoparticles, immune-mediated pulmonary inflammation, lipopolysaccharide, ovalbumin, elastase

## Abstract

Although the adverse health effects of nanoparticles have been proposed and are being clarified, their aggravating effects on pre-existing pathological conditions have not been fully investigated. In this review, we provide insights into the immunotoxicity of both airborne and engineered nanoparticles as an exacerbating factor on hypersusceptible subjects, especially those with immune-mediated pulmonary inflammation, using our *in vivo* experimental model. First, we exhibit the effects of nanoparticles on pulmonary inflammation induced by bacterial endotoxin (lipopolysaccharide: LPS) as a disease model in innate immunity, and demonstrate that nanoparticles instilled through both an intratracheal tube and an inhalation system can exacerbate the lung inflammation. Second, we introduce the effects of nanoparticles on allergic pulmonary inflammation as a disease model in adaptive immunity, and show that repetitive pulmonary exposure to nanoparticles has aggravating effects on allergic inflammation, including adjuvant effects on Th2-milieu. Third, we show that very small nanoparticle exposure exacerbates emphysematous pulmonary inflammation, which is concomitant with enhanced lung expression of proinflammatory molecules (including those that are innate immunity related). Taken together, nanoparticle exposure may synergistically facilitate pathological pulmonary inflammation via both innate and adaptive immunological impairment.

## INTRODUCTION

Epidemiological studies have demonstrated a correlation between exposure to air pollutant particles at the concentrations currently found in major metropolitan areas and mortality and morbidity[1]. The concentration of particulate matter (PM) with a mass median aerodynamic diameter (a density-dependent unit of measure used to describe the diameter of the particle) ≤2.5 μm ($PM_{2.5}$) is more closely associated with both acute and chronic respiratory effects and subsequent mortality than larger particles of ≤10 μm ($PM_{10}$)[2]. In addition, one intriguing aspect of the epidemiologic data is that health effects of $PM_{2.5}$ are primarily seen in subjects with predisposing factors, including pneumonia, asthma, chronic obstructive pulmonary disease, compromised immune systems, atherosclerosis, age over 65 years, and possible depressive state[3,4,5,6]. Partially consistent with the epidemiological studies, we and others have experimentally demonstrated that diesel exhaust particles (DEP), major contributors to environmental $PM_{2.5}$ in urban areas, exhibit respiratory toxicity with or without predisposing pathologies, including allergic asthma, pulmonary emphysema, and acute renal failure (although we did not provide evidence that DEP significantly exacerbated a murine model of pulmonary emphysema in our system[7]) *in vivo*[8,9,10,11,12,13,14,15,16].

To date, nanoparticles, particles <0.1 μm in mass median aerodynamic diameter, have been shown to be increasing in ambient air[17]. Recent measurements indicate that nanoparticle numbers in ambient air range from $2 \times 10^4$/cm$^3$ to $2 \times 10^5$/cm$^3$, with mass concentrations of >50 μg/m$^3$ near major highways[18,19]. On the other hand, nanotechnology is now advancing at an incredible pace, such that it is has created an alternative industrial revolution over the past few years[20]. Consistent with this, the use of engineered nanoparticles (called as "nanomaterials") has been rapidly increasing in commercial applications. As these particles/materials have become more widespread, many questions have arisen regarding the adverse effects they may have on the environment as alternative inhalable toxicants. In particular, due to their sizes, characteristics, and/or existing pattern, nanoparticles/nanomaterials have been implicated in cardiopulmonary system effects[21]. Furthermore, compared to larger particles, nanoparticles/nanomaterials have a higher deposition rate in the peripherial lung, they can cross the pulmonary epithelium and reach the interstitium[22], and may thus be systemically distributed in the bloodstream[23], raising the possibility to render larger degree of inflammation. Nanoparticle/ nanomaterials have an enhanced capacity to produce reactive oxygen species (ROS) and, consequently, have a widespread toxicity[24,25,26], as do other types of PM (with relatively larger particle size), since ROS generation by particles can exert protein, lipid, and membrane damage[27,28]. For example, transition metals and redox-cycling organic chemical components on the particle surface can also participate in ROS generation characterized by the formation of $O_2^-$ through dismutation or Fenton reaction[28]. Indeed, it has been reported that nanomaterial exposure itself induces lung inflammation as for carbon nanotube and titanium dioxide, gold, and quantum dot[29,30,31,32,33,34]. These particle/material exposures also reportedly influence/promote cardiopulmonary systems in the presence of predisposing diseases in human studies[35,36]. However, biological evidence concerning the promoting effects of nanoparticles on predisposing subjects has been less studied. Besides their toxic property on health, therefore, it should be experimentally ascertained whether they also aggravate pre-existing pathological conditions and their underlying mechanisms should be resolved. In this review, we will discuss the impact of nanoparticles as immunological enhancers in the respiratory system.

## EFFECTS OF NANOPARTICLES ON BACTERIAL ENDOTOXIN-ELICITED PULMONARY INFLAMMATION

A glycolipid of Gram-negative bacteria, known as endotoxin or lipopolysaccharide (LPS), stimulates host cells via innate immunity[37]. In animal models, intratracheal administration of LPS causes lung cytokine expression, neutrophil recruitment, and lung injury[38]. LPS is found in the bronchoalveolar lavage (BAL) fluid of patients with pneumonia[39] and acute respiratory distress syndrome[40], which sometimes results in a fatal outcome. In addition, LPS is a significant constituent of many air pollutant particles and has accordingly been implicated in the adverse effects of PM[41]. In accordance with the close links among LPS, lung inflammation (injury), and PM, we have previously shown that intratracheal administration of DEP and their components facilitates lung inflammation induced by LPS[14,42].

We previously examined the effects of pulmonary exposure to nanoparticles on lung inflammation related to LPS in mice. Vehicle, two different types (particle size: 14 and 56 nm) of carbon black nanoparticles (CBNP), LPS, or LPS + CBNP was administered intratracheally, and parameters of lung inflammation and coagulation were evaluated. CBNP alone induced slight lung inflammation and significant pulmonary edema as compared with the vehicle. The 14-nm CBNP intensively aggravated

LPS-elicited lung inflammation and pulmonary edema, which was concomitant with the enhanced lung expression of interleukin (IL)-1β, macrophage inflammatory protein (MIP)-1α, macrophage chemoattractant protein (MCP)-1, MIP-2, and keratinocyte chemoattractant (KC) in overall trend, whereas the 56-nm CBNP did not show apparent effects. Immunoreactivity for 8-hydroxyguanosine (OHdG), a proper marker for oxidative stress, was more intense in the lung from the LPS + 14-nm CBNP group than in that from the LPS group. Taken together, CBNP can aggravate lung inflammation related to bacterial endotoxin, which is more prominent with smaller particles. The enhancing effect may be mediated, at least partly, via the increased local expression of proinflammatory cytokines and via the oxidative stress[43].

Furthermore, we examined the adverse effects of engineered nanoparticles on this pathological model. In brief, Institute for Cancer Research (ICR) male mice were divided into eight experimental groups that intratracheally received vehicle, three sizes (15, 50, or 100 nm) of $TiO_2$ nanoparticles, LPS, or LPS + $TiO_2$ nanoparticles. Twenty-four hours after the treatment, this type of nanoparticle exacerbated the lung inflammation and edema elicited by LPS, with an overall trend of amplified lung expressions of cytokines, such as IL-1β, MCP-1, and KC. LPS + nanoparticles, especially with size <50 nm, elevated circulatory levels of IL-1β, MCP-1, and KC as compared with LPS alone. The enhancement tended overall to be greater with the smaller nanoparticles than with the larger ones. These results suggest that engineered nanoparticles also exacerbate lung inflammation related to LPS and accompanying systemic inflammation, and the exacerbation is more prominent with smaller nanoparticles than with larger particles[44]. Additionally, we demonstrated that latex nanoparticles[45] and carbon nanotubes[46] have similar aggravating potential on the lung pathophysiology.

Our next study was conducted to determine whether inhaled exposure to diesel engine–derived nanoparticles also exacerbates the model. ICR mice were exposed for 5 h to clean air or diesel engine–derived nanoparticles at a concentration of 15, 36, or 169 $\mu g/m^3$ after intratracheal challenge with LPS or vehicle, and were sacrificed for evaluation 24 h after the intratracheal challenge. Exposure to nanoparticles alone did not elicit lung inflammation. Nanoparticle inhalation exaggerated LPS-elicited inflammatory cell recruitment in the BAL fluid and lung parenchyma as compared with clean air inhalation in a concentration-dependent manner. Lung homogenates derived from the LPS + nanoparticle groups tended to have an increased tumor necrosis factor-α level and chemotaxis activity for polymorphonuclear leukocytes as compared to those from the LPS group or the corresponding nanoparticle groups. Nanoparticle inhalation did not significantly increase the lung expression of proinflammatory cytokines or influence systemic inflammation. Isolated alveolar macrophages from nanoparticle-exposed mice showed a greater production of IL-1β and KC stimulated with *ex vivo* LPS challenge than those from clean air–exposed mice, although the differences did not reach significance. These results suggest that acute exposure to diesel nanoparticles exacerbates lung inflammation induced by LPS[47]. In sum, nanoparticle exposure exacerbates acute lung inflammation related to bacterial endotoxin (Fig. 1).

## EFFECTS OF NANOPARTICLES ON ALLERGEN-ELICITED PULMONARY INFLAMMATION

Bronchial asthma has been recognized as chronic airway inflammation with hyper-responsiveness that is characterized by an increase in the number of activated lymphocytes and eosinophils[48]. A number of studies have shown that various particles, including carbon black, can enhance allergic sensitization[49,50,51], which is referred to as "adjuvant effect". As well, carbon black has been demonstrated to enhance the proliferation of antibody-forming cells and both IgE and IgG levels[52,53]. Ultrafine particles (PM and carbon black) reportedly exaggerate allergic airway inflammation *in vivo*[54,55]. However, all studies have failed to pay attention to the size of the particles. Therefore, no research has addressed the size effects of particles or nanoparticles on airway biology in the presence or absence of allergen *in vivo*. Given the hypothesis, we investigated the effects of CBNP with a diameter of 14 or 56 nm on allergen-related airway inflammation. ICR mice were divided into six experimental groups.

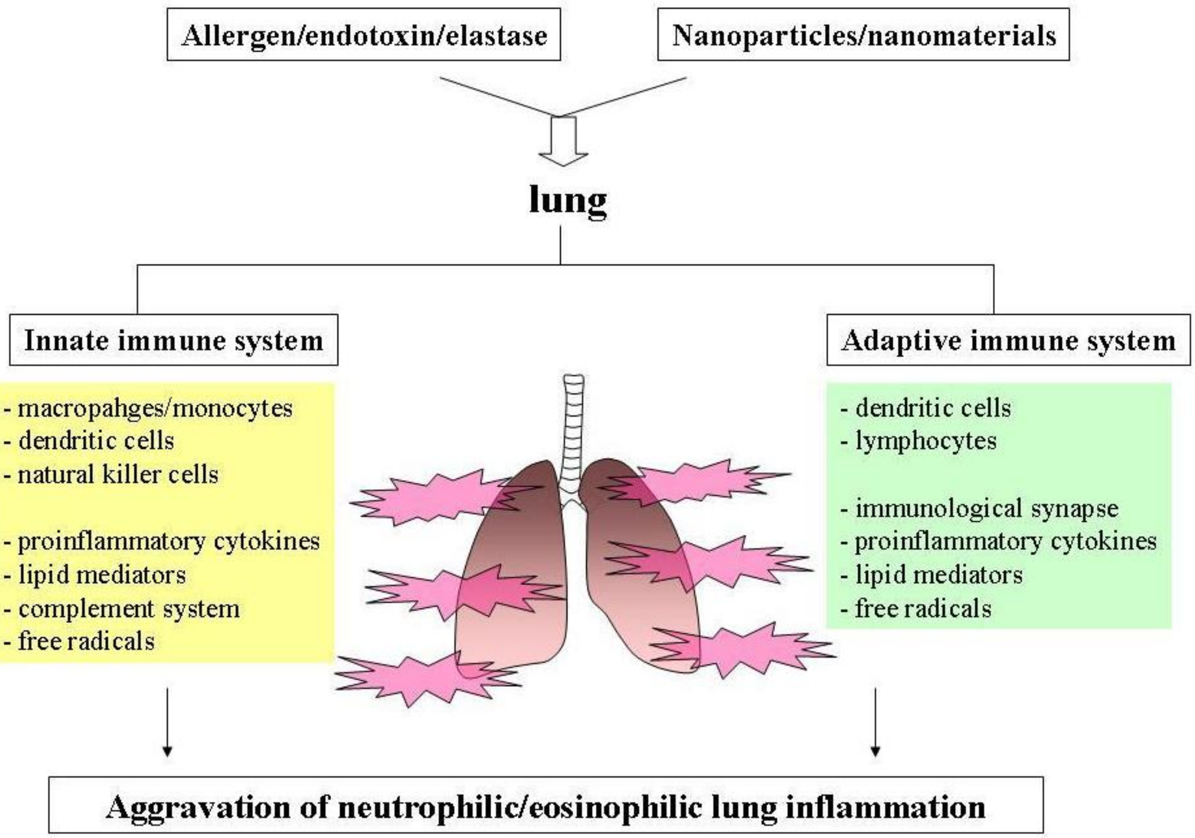

**FIGURE 1.** Proposal schema for aggravating effects of nanoparticles/nanomaterials on immune-mediated pulmonary inflammation. Regarding the impact on innate immunity, these particles may directly/indirectly influence on related cell populations, such as macrophages/monocytes, neutrophils, dendritic cells, natural killer cells, etc. As for adaptive immunity, in turn, nanoparticles/nanomaterials may potentially activate dendritic cells, lymphocytes, eosinophils, and mast cells/basophils. Furthermore, cell-cell interaction, intracellular signaling pathways, and/or chemical mediators, such as proinflammatory cytokines, complement system, and lipid mediators, may also be targets for these particles.

Vehicle, two sizes of CBNP, ovalbumin (OVA), and OVA + CBNP were administered intratracheally. The cellular profile of BAL fluid; lung histology; expression of cytokines, chemokines, and 8-OHdG; and immunoglobulin production were studied. CBNP with a diameter of 14 or 56 nm aggravated allergen-related airway inflammation characterized by the infiltration of eosinophils, neutrophils, and mononuclear cells, and by an increase in the number of goblet cells in the bronchial epithelium. CBNP with allergen increased protein levels of IL-5, IL-6, IL-13, eotaxin, MCP-1, and RANTES (regulated on activation and normal T cells expressed and secreted) in the lung as compared with allergen alone. The formation of 8-OHdG was moderately induced by CBNP or allergen alone, and was markedly enhanced by allergen plus CBNP as compared with CBNP or allergen alone. The aggravation was more prominent with 14-nm CBNP than with 56-nm CBNP in terms of the overall trend. CBNP with a diameter of 14 nm exhibited adjuvant activity for total IgE and antigen-specific IgG and IgE. CBNP can aggravate allergen-related airway inflammation and immunoglobulin production, which becomes more prominent with smaller particles. The enhancement may be mediated, at least partly, by the increased local expression of IL-5 and eotaxin, and also by the modulated expression of IL-13, RANTES, MCP-1, and IL-6[56]. Consistent with our study, de Haar and colleagues have previously shown that nanoparticles (14 and 29 nm) potently facilitate allergic airway inflammation as compared to fine particles (250 and 260 nm)[57].

In ongoing reports, CBNP alone or OVA alone moderately enhanced cholinergic airway reactivity, as assessed by total respiratory system resistance (R) and Newtonian resistance ($R_n$). All the parameters of lung responsiveness, such as R, compliance, elastance, $R_n$, tissue damping, and tissue elastance, were worse in the OVA + CBNP groups than in the vehicle group, the corresponding CBNP groups, or the OVA group. The lung mRNA level for mucin (Muc)5ac was significantly higher in the OVA group than in the vehicle group, and further increased in the OVA + CBNP groups than in the OVA or CBNP groups. These data suggest that CBNP can facilitate lung physiology, such as airway hyper-responsiveness, especially in the presence of allergen. Further, the effects may be mediated, at least partly, through the enhanced lung expression of Muc5ac[58].

We recently demonstrated that (single- and multiwalled) carbon nanotubes promote allergic airway inflammation in mice, which may be partly through enhanced oxidative stress in the airway and the inappropriate activation of antigen-presenting cells, including dendritic cells (*in vitro*)[59,60]. In addition, other groups have reported the similar impacts of nanoparticles (carbon nanotubes, $TiO_2$, gold) as we did on animal allergic asthma models[57,61,62,63]. Moreover, as for cellular contribution, we and others have claimed that antigen-presenting cells, such as dendritic cells, are important target cell populations for the adjuvant activity of nanoparticles[64,65,66]. Taken together, nanoparticle exposure can exacerbate allergic asthma (Fig. 1).

## EFFECTS OF NANOPARTICLES ON ELASTASE-ELICITED PULMONARY INFLAMMATION

Chronic obstructive pulmonary disease (COPD) is one of the chronic inflammatory diseases of the lung that is associated with reduced maximal expiratory flow, increased lung volume, and alveolar wall destruction[67]. COPD is currently the fourth leading cause of death in the U.S., with up to 7 million patients diagnosed each year[68]. PM is epidemiologically implicated to link to the degree of symptoms of COPD[69,70,71]; however, biological evidence remains unclarified. We recently investigated the impact of pulmonary exposure to CBNP on COPD-like emphysematous lung injury induced by porcine pancreatic elastase (PPE) in mice. Vehicle, two sizes (14 and 56 nm) of CBNP, PPE, or PPE + CBNP was administered intratracheally; thereafter, parameters of inflammatory lung changes were evaluated at several time points. CBNP of 14 nm significantly induced acute lung inflammation in nonelicited subjects, and aggravated PPE-elicited airway inflammation with infiltrated neutrophils and eosinophils at an early stage, which was concomitant with the enhanced lung expression of proinflammatory cytokines related to innate immunity, such as IL-1β, and chemokines, such as KC. Further, 14-nm CBNP exaggerated emphysematous lung structural changes at a delayed stage. On the other hand, 56-nm CBNP induced lung inflammation, but did not influence PPE-elicited pathophysiologic traits in the lung. Taken together, CBNP at an optimal size and dose can exacerbate PPE-induced pulmonary inflammatory response and emphysema. This enhancement may be mediated, at least partly, via the increased local expression of proinflammatory molecules([72], and unpublished data) (Fig. 1).

## SUMMARY

In summary, both airborne and engineered nanoparticles/nanomaterials can facilitate pathological pulmonary inflammation, sometimes with synergism, implying that particle size plays a critical role in determining the extent of these types of immune-mediated pulmonary inflammation. Regarding the impact on innate immunity, these particles may directly/indirectly influence on related cell populations, such as macrophages/monocytes, neutrophils, dendritic cells, natural killer cells, etc. As for adaptive immunity, in turn, nanoparticles/nanomaterials may potentially activate dendritic cells, lymphocytes, eosinophils, and mast cells/basophils. Furthermore, cell-cell interaction, intracellular signaling pathways, and/or chemical mediators, such as proinflammatory cytokines, complement system, and lipid mediators, may also be targets for these particles (Fig. 1). Although it is easy to imagine that characteristics of the particles, such as physicochemical properties, electronic charge, aggregation rate, surface coating, colloidal stability, etc., are important for differences in the impact on immune modulation, future investigation warrants the matter. In any case, these previous findings indicate that nanoparticles/ nanomaterials potentiate an inflammatory response in subjects with lung inflammation, which might play a vital role in the pulmonary effects of airborne pollutants on the sensitive populations who have preceding respiratory immune-mediated diseases.

## ACKNOWLEDGMENT

Special thanks to Prof. Takamichi Ichinose, Prof. Seishiro Hirano, Dr. Rie Yanagisawa, and Dr. Eiko Koike for greatly contributing to the work in the manuscript. The authors are supported by NIES grants and partly by Grants-in-Aid for Scientific Research (B) 18390188 (to K. Inoue) from the Japan Society for the Promotion of Science.

## References

1. Samet, J.M., Dominici, F., Curriero, F.C., Coursac, I., and Zeger, S.L. (2000) Fine particulate air pollution and mortality in 20 U.S. cities, 1987-1994. *N. Engl. J. Med.* **343,** 1742–1749.
2. Peters, A., Wichmann, H.E., Tuch, T., Heinrich, J., and Heyder, J. (1997) Respiratory effects are associated with the number of ultrafine particles. *Am. J. Respir. Crit. Care Med.* **155,** 1376–1383.
3. Szyszkowicz, M. (2007) Air pollution and emergency department visits for depression in Edmonton, Canada. *Int. J. Occup. Med. Environ. Health* **20,** 241–245.
4. Kunzli, N., Bridevaux, P.O., Liu, L.J., Garcia-Esteban, R., Schindler, C., Gerbase, M.W., Sunyer, J., Keidel, D., and Rochat, T. (2009) Traffic-related air pollution correlates with adult-onset asthma among never-smokers. *Thorax* **64,** 664–670.
5. Neupane, B., Jerrett, M., Burnett, R.T., Marrie, T., Arain, A., and Loeb, M. (2010) Long-term exposure to ambient air pollution and risk of hospitalization with community-acquired pneumonia in older adults. *Am. J. Respir. Crit. Care Med.* **181,** 47–53.
6. Dockery, D.W., Pope, C.A., 3rd, Xu, X., Spengler, J.D., Ware, J.H., Fay, M.E., Ferris, B.G., Jr., and Speizer, F.E. (1993) An association between air pollution and mortality in six U.S. cities. *N. Engl. J. Med.* **329,** 1753–1759.
7. Inoue, K., Koike, E., and Takano, H. (2010) Comprehensive analysis of elastase-induced pulmonary emphysema in mice: effects of ambient existing particulate matters. *Int. Immunopharmacol.* **10,** 1380–1389.
8. Nemmar, A., Al-Salam, S., Zia, S., Yasin, J., Al Husseni, I., and Ali, B.H. (2010) Diesel exhaust particles in the lung aggravate experimental acute renal failure. *Toxicol. Sci.* **113,** 267–277.
9. Lopes, F.D., Pinto, T.S., Arantes-Costa, F.M., Moriya, H.T., Biselli, P.J., Ferraz, L.F., Lichtenfels, A.J., Saldiva, P.H., Mauad, T., and Martins, M.A. (2009) Exposure to ambient levels of particles emitted by traffic worsens emphysema in mice. *Environ. Res.* **109,** 544–551.
10. Inoue, K., Takano, H., Yanagisawa, R., Ichinose, T., Shimada, A., and Yoshikawa, T. (2005) Pulmonary exposure to diesel exhaust particles induces airway inflammation and cytokine expression in NC/Nga mice. *Arch. Toxicol.* **79,** 595–599.
11. Ichinose, T., Furuyama, A., and Sagai, M. (1995) Biological effects of diesel exhaust particles (DEP). II. Acute toxicity of DEP introduced into lung by intratracheal instillation. *Toxicology* **99,** 153–167.
12. Ichinose, T., Yajima, Y., Nagashima, M., Takenoshita, S., Nagamachi, Y., and Sagai, M. (1997) Lung carcinogenesis and formation of 8-hydroxy-deoxyguanosine in mice by diesel exhaust particles. *Carcinogenesis* **18,** 185–192.
13. Takano, H., Yoshikawa, T., Ichinose, T., Miyabara, Y., Imaoka, K., and Sagai, M. (1997) Diesel exhaust particles enhance antigen-induced airway inflammation and local cytokine expression in mice. *Am. J. Respir. Crit. Care Med.* **156,** 36–42.
14. Takano, H., Yanagisawa, R., Ichinose, T., Sadakane, K., Yoshino, S., Yoshikawa, T., and Morita, M. (2002) Diesel exhaust particles enhance lung injury related to bacterial endotoxin through expression of proinflammatory cytokines, chemokines, and intercellular adhesion molecule-1. *Am. J. Respir. Crit. Care Med.* **165,** 1329–1335.
15. Inoue, K., Takano, H., Sakurai, M., Oda, T., Tamura, H., Yanagisawa, R., Shimada, A., and Yoshikawa T. (2006) Pulmonary exposure to diesel exhaust particles enhances coagulatory disturbance with endothelial damage and systemic inflammation related to lung inflammation. *Exp. Biol. Med. (Maywood)* **231,** 1626–1632.

16. Inoue, K., Koike, E., Takano, H., Yanagisawa, R., Ichinose, T., and Yoshikawa, T. (2009) Effects of diesel exhaust particles on antigen-presenting cells and antigen-specific Th immunity in mice. *Exp. Biol. Med. (Maywood)* **234,** 200–209.
17. Cyrys, J., Stolzel, M., Heinrich, J., Kreyling, W.G., Menzel, N., Wittmaack, K., Tuch, T., and Wichmann, H.E. (2003) Elemental composition and sources of fine and ultrafine ambient particles in Erfurt, Germany. *Sci. Total Environ.* **305,** 143–156.
18. Timonen, K.L., Hoek, G., Heinrich, J., Bernard, A., Brunekreef, B., de Hartog, J., Hameri, K., Ibald-Mulli, A., Mirme, A., Peters, A., Tiittanen, P., Kreyling, W.G., and Pekkanen, J. (2004) Daily variation in fine and ultrafine particulate air pollution and urinary concentrations of lung Clara cell protein CC16. *Occup. Environ. Med.* **61,** 908–914.
19. Zhu, Y., Hinds, W.C., Kim, S., and Sioutas, C. (2002) Concentration and size distribution of ultrafine particles near a major highway. *J. Air Waste Manag. Assoc.* **52,** 1032–1042.
20. Service, R.F. (2004) Nanotoxicology. Nanotechnology grows up. *Science* **304,** 1732–1734.
21. Utell, M.J. and Frampton, M.W. (2000) Acute health effects of ambient air pollution: the ultrafine particle hypothesis. *J. Aerosol Med.* **13,** 355–359.
22. Oberdorster, G. (2001) Pulmonary effects of inhaled ultrafine particles. *Int. Arch. Occup. Environ. Health* **74,** 1–8.
23. Seaton, A., MacNee, W., Donaldson, K., and Godden, D. (1995) Particulate air pollution and acute health effects. *Lancet* **345,** 176–178.
24. Brown, D.M., Wilson, M.R., MacNee, W., Stone, V., and Donaldson, K. (2001) Size-dependent proinflammatory effects of ultrafine polystyrene particles: a role for surface area and oxidative stress in the enhanced activity of ultrafines. *Toxicol. Appl. Pharmacol.* **175,** 191–199.
25. Dick, C.A., Brown, D.M., Donaldson, K., and Stone, V. (2003) The role of free radicals in the toxic and inflammatory effects of four different ultrafine particle types. *Inhal. Toxicol.* **15,** 39–52.
26. Li, N., Sioutas, C., Cho, A., Schmitz, D., Misra, C., Sempf, J., Wang, M., Oberley, T., Froines, J., and Nel, A. (2003) Ultrafine particulate pollutants induce oxidative stress and mitochondrial damage. *Environ. Health Perspect.* **111,** 455–460.
27. Nel, A., Xia, T., Madler, L., and Li, N. (2006) Toxic potential of materials at the nanolevel. *Science* **311,** 622–627.
28. Li, N., Xia, T., and Nel, A. (2008) The role of oxidative stress in ambient particulate matter-induced lung diseases and its implications in the toxicity of engineered nanoparticles. *Free Radic. Biol. Med.* **44,** 1689–1699.
29. Warheit, D.B., Laurence, B.R., Reed, K.L., Roach, D.H., Reynolds, G.A., and Webb, T.R. (2004) Comparative pulmonary toxicity assessment of single-wall carbon nanotubes in rats. *Toxicol. Sci.* **77,** 117–125.
30. Shvedova, A.A., Kisin, E.R., Mercer, R., Johnson, V.J., Potapovich, A.I., Tyurina, Y.Y., Gorelik, O., Arepalli, S., Schwegler-Berry, D., Hubbs, A.F., Antonini, J., Evans, D.E., Ku, B.K., Ramsey, D., Maynard, A., Kagan, V.E., Castranova, V., and Baron, P. (2005) Unusual inflammatory and fibrogenic pulmonary responses to single-walled carbon nanotubes in mice. *Am. J. Physiol. Lung Cell. Mol. Physiol.* **289,** L698–708.
31. Chen, H.W., Su, S.F., Chien, C.T., Lin, W.H., Yu, S.L., Chou, C.C., Chen, J.J., and Yang, P.C. (2006) Titanium dioxide nanoparticles induce emphysema-like lung injury in mice. *FASEB J.* **20,** 2393–2395.
32. Warheit, D.B., Webb, T.R., Sayes, C.M., Colvin, V.L., and Reed, K.L. (2006) Pulmonary instillation studies with nanoscale TiO2 rods and dots in rats: toxicity is not dependent upon particle size and surface area. *Toxicol. Sci.* **91,** 227–236.
33. Gosens, I., Post, J.A., de la Fonteyne, L.J., Jansen, E.H., Geus, J.W., Cassee, F.R., and de Jong, W.H. (2010) Impact of agglomeration state of nano- and submicron sized gold particles on pulmonary inflammation. *Part. Fibre. Toxicol.* **7,** 37.
34. Jacobsen, N.R., Moller, P., Jensen, K.A., Vogel, U., Ladefoged, O., Loft, S., and Wallin, H. (2009) Lung inflammation and genotoxicity following pulmonary exposure to nanoparticles in ApoE-/- mice. *Part. Fibre. Toxicol.* **6,** 2.
35. Gong, H., Jr., Linn, W.S., Clark, K.W., Anderson, K.R., Sioutas, C., Alexis, N.E., Cascio, W.E., and Devlin, R.B. (2008) Exposures of healthy and asthmatic volunteers to concentrated ambient ultrafine particles in Los Angeles. *Inhal. Toxicol.* **20,** 533–545.
36. Frampton, M.W., Stewart, J.C., Oberdorster, G., Morrow, P.E., Chalupa, D., Pietropaoli, A.P., Frasier, L.M., Speers, D.M., Cox, C., Huang, L.S., and Utell, M.J. (2006) Inhalation of ultrafine particles alters blood leukocyte expression of adhesion molecules in humans. *Environ. Health Perspect.* **114,** 51–58.
37. Vincenti, M.P., Burrell, T.A., and Taffet, S.M. (1992) Regulation of NF-kappa B activity in murine macrophages: effect of bacterial lipopolysaccharide and phorbol ester. *J. Cell. Physiol.* **150,** 204–213.
38. Ulich, T.R., Watson, L.R., Yin, S.M., Guo, K.Z., Wang, P., Thang, H., and del Castillo, J. (1991) The intratracheal administration of endotoxin and cytokines. I. Characterization of LPS-induced IL-1 and TNF mRNA expression and the LPS-, IL-1-, and TNF-induced inflammatory infiltrate. *Am. J. Pathol.* **138,** 1485–1496.
39. Flanagan, P.G., Jackson, S.K., and Findlay, G. (2001) Diagnosis of gram negative, ventilator associated pneumonia by assaying endotoxin in bronchial lavage fluid. *J. Clin. Pathol.* **54,** 107–110.

40. Martin, T.R., Rubenfeld, G.D., Ruzinski, J.T., Goodman, R.B., Steinberg, K.P., Leturcq, D.J., Moriarty, A.M., Raghu, G., Baughman, R.P., and Hudson, L.D. (1997) Relationship between soluble CD14, lipopolysaccharide binding protein, and the alveolar inflammatory response in patients with acute respiratory distress syndrome. *Am. J. Respir. Crit. Care Med.* **155,** 937–944.
41. Becker, S., Fenton, M.J., and Soukup, J.M. (2002) Involvement of microbial components and toll-like receptors 2 and 4 in cytokine responses to air pollution particles. *Am. J. Respir. Cell Mol. Biol.* **27,** 611–618.
42. Yanagisawa, R., Takano, H., Inoue, K., Ichinose, T., Sadakane, K., Yoshino, S., Yamaki, K., Kumagai, Y., Uchiyama, K., Yoshikawa, T., and Morita, M. (2003) Enhancement of acute lung injury related to bacterial endotoxin by components of diesel exhaust particles. *Thorax* **58,** 605–612.
43. Inoue, K., Takano, H., Yanagisawa, R., Hirano, S., Sakurai, M., Shimada, A., and Yoshikawa, T. (2006) Effects of airway exposure to nanoparticles on lung inflammation induced by bacterial endotoxin in mice. *Environ. Health Perspect.* **114,** 1325–1330.
44. Inoue, K., Takano, H., Ohnuki, M., Yanagisawa, R., Sakurai, M., Shimada, A., Mizushima, K., and Yoshikawa, T. (2008) Size effects of nanomaterials on lung inflammation and coagulatory disturbance. *Int. J. Immunopathol. Pharmacol.* **21,** 197–206.
45. Inoue, K., Takano, H., Yanagisawa, R., Koike, E., and Shimada, A. (2009) Size effects of latex nanomaterials on lung inflammation in mice. *Toxicol. Appl. Pharmacol.* **234,** 68–76.
46. Inoue, K., Takano, H., Koike, E., Yanagisawa, R., Sakurai, M., Tasaka, S., Ishizaka, A., and Shimada, A. (2008) Effects of pulmonary exposure to carbon nanotubes on lung and systemic inflammation with coagulatory disturbance induced by lipopolysaccharide in mice. *Exp. Biol. Med. (Maywood)* **233,** 1583–1590.
47. Inoue, K.I., Takano, H., Yanagisawa, R., Hirano, S., Kobayashi, T., Fujitani, Y., Shimada, A., and Yoshikawa, T. (2007) Effects of inhaled nanoparticles on acute lung injury induced by lipopolysaccharide in mice. *Toxicology* **238,** 99–110.
48. Agrawal, D.K. and Shao, Z. (2010) Pathogenesis of allergic airway inflammation. *Curr. Allergy Asthma Rep.* **10,** 39–48.
49. Maejima, K., Tamura, K., Taniguchi, Y., Nagase, S., and Tanaka H. (1997) Comparison of the effects of various fine particles on IgE antibody production in mice inhaling Japanese cedar pollen allergens. *J. Toxicol. Environ. Health* **52,** 231–248.
50. Lambert, A.L., Dong, W., Winsett, D.W., Selgrade, M.K., and Gilmour, M.I. (1999) Residual oil fly ash exposure enhances allergic sensitization to house dust mite. *Toxicol. Appl. Pharmacol.* **158,** 269–277.
51. Lambert, A.L., Dong, W., Selgrade, M.K., and Gilmour, M.I. (2000) Enhanced allergic sensitization by residual oil fly ash particles is mediated by soluble metal constituents. *Toxicol. Appl. Pharmacol.* **165,** 84–93.
52. Lovik, M., Hogseth, A.K., Gaarder, P.I., Hagemann, R., and Eide, I. (1997) Diesel exhaust particles and carbon black have adjuvant activity on the local lymph node response and systemic IgE production to ovalbumin. *Toxicology* **121,** 165–178.
53. van Zijverden, M., van der Pijl, A., Bol, M., van Pinxteren, F.A., de Haar, C., Penninks, A.H., van Loveren, H., and Pieters, R. (2000) Diesel exhaust, carbon black, and silica particles display distinct Th1/Th2 modulating activity. *Toxicol. Appl. Pharmacol.* **168,** 131–139.
54. Last, J.A., Ward, R., Temple, L., Pinkerton, K.E., and Kenyon, N.J. (2004) Ovalbumin-induced airway inflammation and fibrosis in mice also exposed to ultrafine particles. *Inhal. Toxicol.* **16,** 93–102.
55. Al-Humadi, N.H., Siegel, P.D., Lewis, D.M., Barger, M.W., Ma, J.Y., Weissman, D.N., and Ma, J.K. (2002) The effect of diesel exhaust particles (DEP) and carbon black (CB) on thiol changes in pulmonary ovalbumin allergic sensitized Brown Norway rats. *Exp. Lung Res.* **28,** 333–349.
56. Inoue, K., Takano, H., Yanagisawa, R., Sakurai, M., Ichinose, T., Sadakane, K., and Yoshikawa, T. (2005) Effects of nano particles on antigen-related airway inflammation in mice. *Respir. Res.* **6,** 106.
57. de Haar, C., Hassing, I., Bol, M., Bleumink, R., and Pieters, R. (2006) Ultrafine but not fine particulate matter causes airway inflammation and allergic airway sensitization to co-administered antigen in mice. *Clin. Exp. Allergy* **36,** 1469–1479.
58. Inoue, K., Takano, H., Yanagisawa, R., Sakurai, M., Abe, S., Yoshino, S., Yamaki, K., and Yoshikawa, T. (2007) Effects of nanoparticles on lung physiology in the presence or absence of antigen. *Int. J. Immunopathol. Pharmacol.* **20,** 737–744.
59. Inoue, K., Koike, E., Yanagisawa, R., Hirano, S., Nishikawa, M., and Takano, H. (2009) Effects of multi-walled carbon nanotubes on a murine allergic airway inflammation model. *Toxicol. Appl. Pharmacol.* **237,** 306–316.
60. Inoue, K., Yanagisawa, R., Koike, E., Nishikawa, M., and Takano, H. (2010) Repeated pulmonary exposure to single-walled carbon nanotubes exacerbates allergic inflammation of the airway: possible role of oxidative stress. *Free Radic. Biol. Med.* **48,** 924–934.
61. Nygaard, U.C., Hansen, J.S., Samuelsen, M., Alberg, T., Marioara, C.D., and Lovik, M. (2009) Single-walled and multi-walled carbon nanotubes promote allergic immune responses in mice. *Toxicol. Sci.* **109,** 113–123.
62. Ryman-Rasmussen, J.P., Tewksbury, E.W., Moss, O.R., Cesta, M.F., Wong, B.A., and Bonner, J.C. (2009) Inhaled multiwalled carbon nanotubes potentiate airway fibrosis in murine allergic asthma. *Am. J. Respir. Cell Mol. Biol.* **40,** 349–358.

63. Hussain, S., Vanoirbeek, J.A., Luyts, K., De Vooght, V., Verbeken, E., Thomassen, L.C., Martens, J.A., Dinsdale, D., Boland, S., Marano, F., Nemery, B., and Hoet, P.H. (2010) Lung exposure to nanoparticles modulates an asthmatic response in a mouse model of asthma. *Eur. Respir. J.* [Epub ahead of print]
64. Koike, E., Takano, H., Inoue, K., Yanagisawa, R., and Kobayashi, T. (2008) Carbon black nanoparticles promote the maturation and function of mouse bone marrow-derived dendritic cells. *Chemosphere* **73,** 371–376.
65. Koike, E., Takano, H., Inoue, K., Yanagisawa, R., Sakurai, M., Aoyagi, H., Shinohara, R., and Kobayashi, T. (2008) Pulmonary exposure to carbon black nanoparticles increases the number of antigen-presenting cells in murine lung. *Int. J. Immunopathol. Pharmacol.* **21,** 35–42.
66. de Haar, C., Kool, M., Hassing, I., Bol, M., Lambrecht, B.N, and Pieters, R. (2008) Lung dendritic cells are stimulated by ultrafine particles and play a key role in particle adjuvant activity. *J. Allergy Clin. Immunol.* **121,** 1246–1254.
67. Pauwels, R.A., Buist, A.S., Calverley, P.M., Jenkins, C.R., and Hurd, S.S. (2001) Global strategy for the diagnosis, management, and prevention of chronic obstructive pulmonary disease. NHLBI/WHO Global Initiative for Chronic Obstructive Lung Disease (GOLD) Workshop summary. *Am. J. Respir. Crit. Care Med.* **163,** 1256–1276.
68. Croxton, T.L., Weinmann, G.G., Senior, R.M., and Hoidal, J.R. (2002) Future research directions in chronic obstructive pulmonary disease. *Am. J. Respir. Crit. Care Med.* **165,** 838–844.
69. Zanobetti, A., Bind, M.A., and Schwartz, J. (2008) Particulate air pollution and survival in a COPD cohort. *Environ. Health* **7,** 48.
70. Torres-Duque, C., Maldonado, D., Perez-Padilla, R., Ezzati, M., and Viegi, G. (2008) Biomass fuels and respiratory diseases: a review of the evidence. *Proc. Am. Thorac. Soc.* **5,** 577–590.
71. Euler, G.L., Abbey, D.E., Magie, A.R., and Hodgkin, J.E. (1987) Chronic obstructive pulmonary disease symptom effects of long-term cumulative exposure to ambient levels of total suspended particulates and sulfur dioxide in California Seventh-Day Adventist residents. *Arch. Environ. Health* **42,** 213–222.
72. Inoue, K., Yanagisawa, R., Koike, E., Ichinose, T., Tasaka, S., Kiyono, M., Nakamura, R., and Takano, H. Effects of carbon black nanoparticles on elastase-induced emphysematous lung injury in mice. *Basic. Clin. Pharmacol. Toxicol.*, in press.
